# Effects of Handedness on Visual Sensitivity in Perihand Space

**DOI:** 10.1371/journal.pone.0043150

**Published:** 2012-08-17

**Authors:** Nathalie Le Bigot, Marc Grosjean

**Affiliations:** Leibniz Research Centre for Working Environment and Human Factors, Dortmund, Germany; Radboud University Nijmegen, Netherlands

## Abstract

Recent studies have shown that changes in visual processing in perihand space are limited to the area around the right hand, at least in right-handers. One explanation for these findings is that perception is altered at locations where action is more likely to occur. To test this notion, we asked both right- and left-handers to perform an unspeeded visual discrimination task under four hand-position configurations: Left hand, right hand, both hands, or no hands near the display. Compared to the no-hands (control) condition, visual sensitivity (*d’*) was higher in the dominant-hand condition for right-handers and higher in the dominant- as well as the non-dominant hand condition for left-handers. When both hands were near the display, sensitivity was similar to that in the dominant-hand condition for right-handers and to that in the non-dominant hand condition for left-handers. This shows that performance differed between the two handedness groups when their non-dominant hand was near the display (both alone and accompanied by their dominant hand). Thus, the pattern for left-handers did not correspond to a mirror image of the pattern for right-handers. In line with studies on bimanual action control, visual processing in perihand space seems to be determined by the different ways in which left- and right-handers use their hands.

## Introduction

There is a growing body of evidence showing that visual processing is altered in perihand space. For example, Reed, Grubb and Steele [1] asked participants to perform a covert visual orienting task [2] while holding one of their hands on one side of the visual display. They observed that target detection was faster for a target appearing on the same side as the hand than for a target appearing on the opposite side. To account for this effect, they proposed that the space near the hand benefits from a form of attentional prioritization that is supported by multimodal representations. Indeed, several observations point to a multisensory coding of objects in peripersonal space, corresponding to the space immediately surrounding the body (e.g., [3]). In particular, neurophysiological studies have revealed that there are bimodal visuo-tactile neurons in various brain areas that respond exclusively to tactile stimulation of a particular body part (e.g., the face or hand) or to visual stimulation near that body part (for a review, see [4]). Thus, visual objects appearing near the hand benefit from bimodal representations that may influence the allocation of visual attention [1].

Since the study by Reed et al. [1], altered visual processing in perihand space has been documented for a variety of attention tasks, including visual search, inhibition of return and the attentional blink [5]. Effects of hand proximity have also been revealed in tasks involving figure-ground assignment [6], change detection [7] or even semantic processing [8]. Critically, a number of control experiments have been performed to ensure that such effects are related to the presence of the hand and not due to an uncomfortable or unusual body position [8], or to the presence of an additional object (i.e., the arm and the hand) in the visual field. For example, it has been repeatedly shown that when the arm and/or hand is replaced with a visual anchor, performance does not differ from situations in which neither hand is near the display [1], [6], [9].

Some recent studies have suggested that the effect of hand proximity is actually limited to the space around the right hand, at least in right-handers. For example, larger spatial cueing effects [10] and better change detection performance [7] have been found when the right, but not the left, hand was positioned near the display. Moreover, this right-hand effect is not restricted to a particular hemispace, as it was still present when participants positioned their right hand within their left hemifield [10]. One explanation of these findings is based on how people interact with their environment [7], [11]. Specifically, right-handers use their right hand more frequently and for more precise actions than their left one [12], making the space around their dominant hand a more functional one. Thus, according to this explanation visual processing is enhanced at locations where action is more likely to occur [13]. Consistent with this notion, Reed et al. ([11], Experiment 1) observed that targets appearing near the palm of the hand (i.e., where one could potentially grasp an object) were detected faster than targets appearing near the back of the hand. They obtained a similar difference for targets presented near the palm of the hand versus near the forearm.

The main goal of the present study was to further test this “functional” hypothesis [7], [11] by exploring the effect of handedness on visual sensitivity in perihand space. Given the differences in how left- and right-handers interact with the environment, they should present different patterns of performance for stimuli presented near their left and right hands. However, the effects of hand proximity for left-handers should not necessarily be reversed relative to those for right-handers, as they differ in how they use their dominant and non-dominant hands [10]. For example, in precision grasping, left-handers are much more likely to use their non-dominant hand than right-handers [12]. This difference was also observed when looking at the lead hand in bimanual coordination tasks. In these studies, the dominant hand was the lead hand for right-handers more often than for left-handers, whether in a bimanual circle drawing task [14] or a bimanual reaction time task to centralized visual stimuli [15].

Another question of interest concerns the level of cognitive processing which is affected by hand proximity. Although certain effects may certainly be considered perceptual in nature, such as changes in figure-ground assignment [6], the level of processing at which they arise has yet to be fully established. For example, hand proximity may have affected perception *per se* or post-perceptual processes, such as decision making, which can also lead to differences in response patterns and reaction times [16], [17], [18]. To establish whether perceptual processing is actually altered in perihand space, it’s important to observe changes in accuracy measures within an experiment that was designed to reveal such effects. These type of experiments generally involve difficult perceptual tasks, by briefly presenting and/or masking the stimuli, and no speed stress which could potentially contaminate accuracy measures [16], [17], [18].

To our knowledge, only two studies have reported hand proximity effects on accuracy measures. Tseng and Bridgeman [7] observed increased change detection performance in perihand space. However, as mentioned by the authors, change detection is a complex task that relies on visual short-term memory and multiple stages of information processing. It is thus impossible to determine at which level of processing hand proximity had an impact. Dufour and Touzalin [9] also reported effects of hand proximity on response accuracy in speeded visual detection and spatial discrimination tasks. Their results revealed greater detection/discrimination accuracy for stimuli that were near compared to far from the hand, while no difference in reaction times was observed. Although the latter finding may indeed reflect a form of visual enhancement in perihand space, the presence of speed stress may have contaminated the accuracy measures and it’s unclear why such perceptual effects didn’t show up in the reaction times as well [16], [17]. In light of these potential limitations, the second goal of the current study was to determine whether hand proximity affects early stages of perceptual processing.

To address this and our main question, we asked left- and right-handers to perform a visual discrimination task under four different hand position configurations: Left hand, right hand, both hands and no hands near the display. We included all four of these positions as previous studies have generally compared a no-hands (control) condition to only a single-hand or both-hands condition (but see [7]). The visual stimuli were presented for a brief individually tracked duration and were always followed by a visual mask. Participants had to discriminate between a target and a distractor by providing an unspeeded “yes”/“no” response that allowed us to obtain an unbiased measure of visual sensitivity (*d’*). Lastly, to avoid potential effects of stimulus-response compatibility (e.g., [19]), we asked participants to provide a non-lateralized oral response. The latter manipulation is of particular importance as hand proximity effects have been found to depend on which effector is used to respond. For example, in their studies on exogenous shifts of attention in perihand space, Llyod et al. [10] obtained different patterns of performance depending on whether people responded with their right (Experiment 1) or left (Experiment 2) foot.

To summarize, if hand proximity enhances early perceptual processing, then visual sensitivity should be higher in perihand space. Moreover, this effect should be modulated by handedness if it’s related to how people use their dominant and non-dominant hands to interact with the environment.

## Methods

### Ethics Statement

The study was conducted according to the code of ethics of the World Medical Association (Declaration of Helsinki) and was approved by the ethics committee of the Leibniz Research Centre of Working Environment and Human Factors. All data were analyzed anonymously and all participants gave written informed consent prior to participation.

### Participants

Fifty-four individuals (mean age = 24.4 years; age range = 20–34 years) took part in the experiment in exchange of course credit or monetary compensation. Six participants were excluded due to floor and/or ceiling effects (see Procedure for details). As assessed with the Edinburgh Handedness Inventory [20], there were 16 left-handers (mean score = –81.88) and 32 right-handers (mean score = 86.63) that remained. All participants had normal or corrected-to-normal vision and were naïve as to the purpose of the study.

### Stimuli and Apparatus

**Figure 1:**
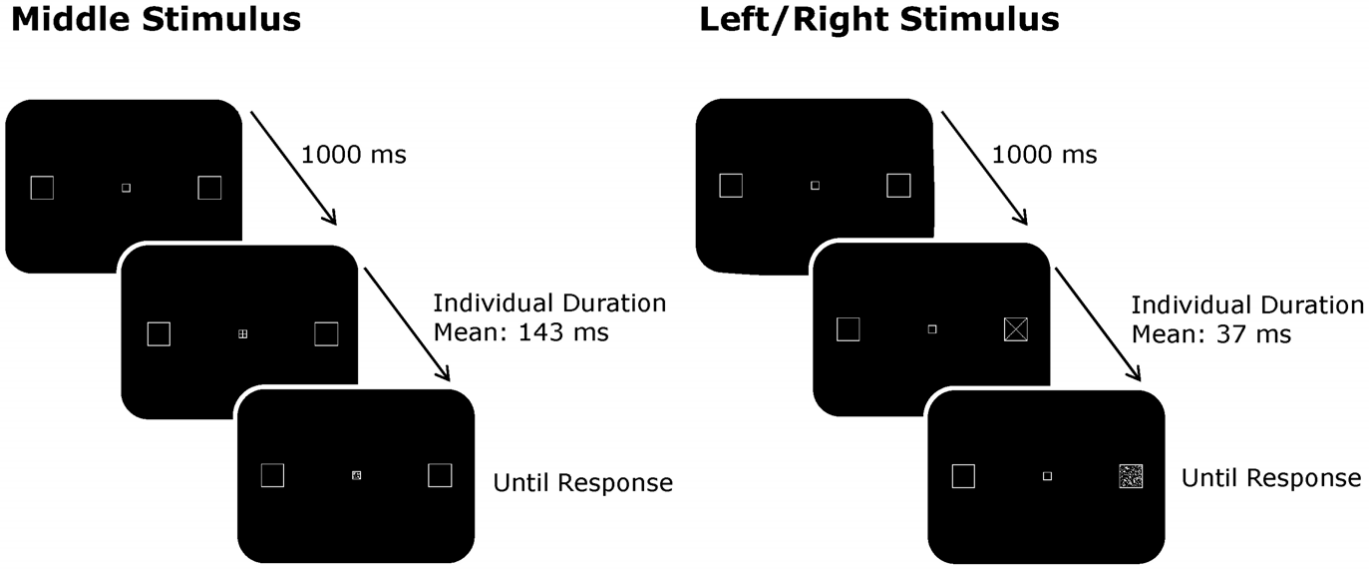
Schematic of the trial events for middle and lateral (left, right) stimulus positions.

Stimuli were presented on a 19-in. monitor (refresh rate: 100 Hz) and viewing distance was held constant at approximately 50 cm by using a chinrest. All stimuli were drawn in grey on a dark background. As illustrated in Figure 1, the fixation display consisted of three empty squares: A middle square (0.56°×0.56° of visual angle) centered in the display and two lateral squares (1.68°×1.68°) located 16.5° (center-to-center) to either side. The lateral squares were larger than the middle one to take cortical magnification into account (e.g., [21]). The next display contained a target (“×” sign) or a distractor (“+” sign) stimulus of matching size within one of the three squares. A random-dot mask (25% density) filled the corresponding square in the final display. Only that location was masked in order to avoid any uncertainty regarding stimulus location.

**Figure 2:**
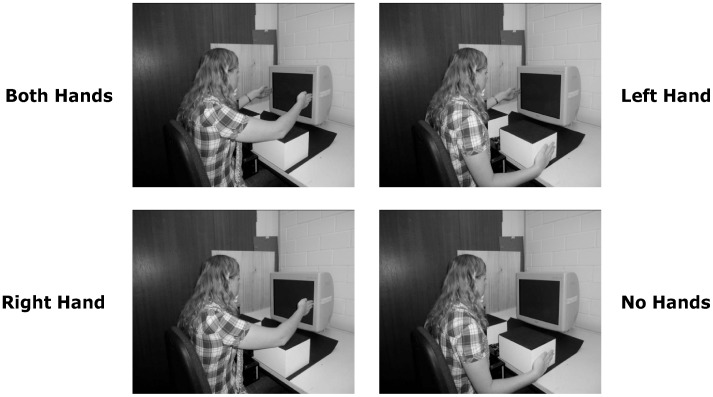
Illustration of the four hand positions (both hands, left hand, right hand, no hands). The person in the photographs has given written informed consent (as outlined in the PLoS consent form) to publication of her image.

As shown in Figure 2, participants performed the task under four different hand-position configurations: With both hands near the sides of the display (both hands), with only their left hand near the left side of the display (left hand), with only their right hand near the right side of the display (right hand), and with neither of their hands near the display (no hands). For hand positions near the display, participants were asked to place their hand close the border of the screen with their middle finger touching it and their palm directed towards the center of the display. In these conditions, the approximate distance between their hand and the different stimulus positions corresponded to 3 cm (4.01°) for a same-side stimulus, 17.5 cm (19.85°) for a middle stimulus, and 32 cm (35.46°) for an opposite-side stimulus. To avoid fatigue, two wooden blocks with adjustable heights were used to support the participants’ arms. For hand positions far from the display, participants were instructed to place their hand on the outer side of the corresponding wooden block (see Figure 2).

### Design

There was one experimental block for each combination of stimulus condition (central: middle position only; lateral: left and right positions) and hand position condition (both hands, left hand, right hand, no hands). Half of the participants performed the first four blocks with central stimuli and then the next four with lateral stimuli; this order was reversed for the other half of participants. The order of hand positions was counterbalanced across participants using a Latin square design and this sequence was repeated for each stimulus condition. In each block, a random half of the trials contained the target and the other half the distractor. When applicable, left and right stimulus positions were used equally often within a block, and targets and distractors were evenly distributed across them. There were 30 trials per block with central stimuli and, because of the doubling of stimulus positions, 60 trials per block with lateral stimuli. This led to a total of 360 experimental trials.

### Procedure

Participants were comfortably seated by adjusting the height of a chair, the chinrest and the wooden blocks for supporting their arms. They were then familiarized with the visual discrimination task by using easily perceived stimulus durations. In experimental blocks, each trial began with the presentation of the fixation display and participants were instructed to focus on the middle square throughout the duration of the trial. After 1000 ms, the stimulus appeared in one of the three squares for an individually set duration (see below) and was then masked until a response was recorded. Participants were asked to report whether the target stimulus had been presented by saying “yes” or “no” and their response was recorded by the experimenter with the help of a computer mouse. Oral responses were used to avoid any effects of spatial stimulus-response compatibility that could arise from responding with one or both of their hands/feet [10]. The screen then went blank and the next trial started 1500 ms later. Participants were provided with a break every 30 trials and the percentage of correct responses was displayed as feedback at the end of each block.

Since the focus of this study was on perceptual performance, no speed stress was placed on responding [16], [17]. Moreover, stimulus durations were adjusted for each participant by employing an adaptive staircase (tracking) procedure to assure that their discrimination performance lay within a sensitive range. Because of differences between foveal and peripheral vision (e.g., [22]) and the blocked nature of the design, tracking was performed in separate blocks for central stimuli (40 trials) and lateral stimuli (80 trials; 40 per stimulus position). These blocks were run in the no-hands condition and prior to beginning the corresponding series of experimental blocks. Stimulus duration started at 150 ms for central stimuli and 200 ms for lateral stimuli. A three-down, one-up algorithm, targeting 79.4% correct, was used [23]. That is, stimulus duration decreased by one step after three successive correct responses and increased by one step after one incorrect response. To speed up the initial part of tracking, step size was at first set to 30 ms and then reduced to 10 ms once the participants had made two incorrect responses. Threshold estimates were obtained by taking the median of the last five stimulus durations used in the tracking. The corresponding mean stimulus durations (across participants) were 37 ms for central stimuli and 143 ms for lateral stimuli.

Despite the use of the adaptive procedure, some participants exhibited floor or ceiling effects in the experimental blocks. Six participants were discarded because they had accuracy rates below 55% or above 95% in at least four of the twelve conditions defined by the three stimulus position and the four hand position conditions. For the remaining participants, mean overall accuracy was 82.4%, which was within the expected range.

## Results

In order to assess visual performance in an unbiased way, perceptual sensitivity (*d’*) and response bias (criterion [*c*]) were computed for each participant and condition using the following formulas [24]: *d’* = *z*(hit) – *z*(false alarm) and *c* = –0.5[*z*(hit) + *z*(false alarm)]. These measures were submitted to separate three-way mixed analyses of variance (ANOVAs) with handedness (left, right) as a between-participants factor and stimulus position (left, middle, right) and hand position (both hands, left hand, right hand, no hands) as within-participant factors. When necessary, violations of the sphericity assumption were corrected for using the Greenhouse-Geisser ε.

**Figure 3:**
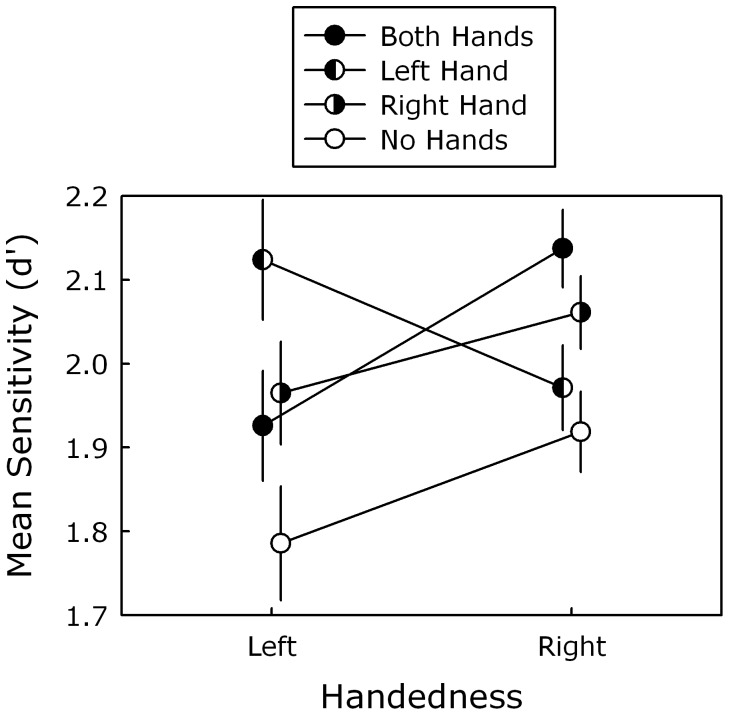
Mean sensitivity (d’) as a function of handedness (left, right) and hand position (both hands, left hand, right hand, no hands). Error bars correspond to ±1 within-participants standard error of the mean [30].

Table 1 presents mean sensitivity as a function of handedness, stimulus position and hand position. The ANOVA on the sensitivity values did not yield a main effect of handedness, *p*>.53, or stimulus position, *p*>.26. Both of these results were to be expected as the adaptive procedure should have equated task difficulty across participants and stimulus positions. More interestingly, there was a significant main effect of hand position, *F*(3, 138) = 3.64, *p*<.05, η^2^ = .07, and this effect was modulated by handedness, as revealed by a significant interaction between these two factors, *F*(3, 138) = 2.75, *p*<.05, η^2^ = .05. As can be seen in Figure 3, which presents mean sensitivity only as a function of handedness and hand position, this interaction reflects that different hand positions led to an enhancement of visual sensitivity in perihand space for left- and right-handers.

**Table 1:** Mean Sensitivity (d’) as a Function of Handedness (Left-Handers, Right-Handers), Stimulus Position (Left, Middle, Right), and Hand Position (Both Hands, Left Hand, Right Hand, No Hands).

	Left-Handers			Right-Handers		
Hand Position	Left	Middle	Right	Left	Middle	Right
Both Hands	1.89	1.87	2.02	1.91	2.19	2.31
Left Hand	2.11	2.22	2.04	1.78	2.13	2.00
Right Hand	1.95	1.94	2.01	1.86	2.13	2.19
No Hands	1.74	1.68	1.94	1.79	2.00	1.97

To further characterize this interaction, planned follow-up tests (one-tailed simple contrasts) were performed comparing perceptual performance in the no-hands (control) condition to the other hand-position conditions. For right-handers, visual sensitivity was significantly higher when both hands, *t*(31) = 3.28, *p<*.01, η^2^ = .26, and only their dominant (right) hand, *t*(31) = 1.85, *p*<.05, η^2^ = .10, were near the display. However, this benefit was absent for the non-dominant (left) hand condition, *p*>.28. The pattern was different for left-handers: Visual sensitivity was significantly higher in the dominant (left) hand condition, *t*(15) = 3.04, *p<*.01, η^2^ = .38, and non-dominant (right) hand condition, *t*(15) = 1.78, *p*<.05, η^2^ = .17, but not in the both-hands condition, *p*>.12, η^2^ = .09. However, the latter result may reflect a slight power problem, as the both-hands condition does not appear to differ from the non-dominant hand condition, which did show an effect (see Figure 3). To further characterize performance in the both-hands condition, additional (two-tailed) comparisons revealed that this condition did not significantly differ from the non-dominant hand condition in left-handers, *p*>.76, nor from the dominant-hand condition in right-handers, *p*>.29.

None of the remaining interactions from the omnibus ANOVA were significant for the sensitivity values, all *p*s >.50. As for response bias (see Table 2), the ANOVA only yielded a significant main effect of stimulus position, *F*(2, 92) = 8.01, *p*<.01, η^2^ = .15, all other *p*s >.17, which reflects that participants responded somewhat more conservatively for central stimuli (*M* = 0.14) than for left and right stimuli (*M* = −0.04 and *M* = 0.02, respectively).

**Table 2:** Mean Bias (c) as a Function of Handedness (Left-Handers, Right-Handers), Stimulus Position (Left, Middle, Right), and Hand Position (Both Hands, Left Hand, Right Hand, No Hands).

	Left-Handers			Right-Handers		
Hand Position	Left	Middle	Right	Left	Middle	Right
Both Hands	−0.03	0.02	0.01	−0.04	0.18	0.06
Left Hand	−0.07	0.11	−0.11	−0.02	0.15	0.15
Right Hand	0.05	0.13	0.00	−0.05	0.17	0.07
No Hands	−0.16	0.15	−0.04	0.02	0.24	−0.01

## Discussion

The purpose of this study was to establish the effect of handedness on visual processing in perihand space. To do so, we tested left- and right-handers on an unspeeded visual-discrimination task designed to assess the influence of hand proximity on perceptual performance in an unbiased fashion. As previous studies have already suggested [7], [9], the current results demonstrate that visual sensitivity is enhanced in near-hand space. Given the nature of the task used here, which involved the brief presentation of masked stimuli and the absence of speed stress, this enhancement probably arises at relatively early stages of perceptual processing, such as by affecting the sensory quality of visual input (for a discussion of this logic, see Introduction and [13], [16]).

The absence of any effects related to stimulus position, however, suggests that the hand-facilitation effect was not limited to the space very close to the hand, at least for the distances used here (up to 32 cm). This result is consistent with previous observations by Tseng and Bridgeman [7], who reported better change detection performance at all of their display locations, regardless of the distance between the hand(s) and the visual change. One explanation for these patterns relates to the notion of object-based attention, which refers to the finding that attention spreads within an object that has been partially cued, rather than only being allocated to the immediate area around the cue [25], [26]. In the same way, the contact of the hand with the screen may have caused the enhancement effect to spread to the whole display. Future research will be needed to elucidate this issue.

Interestingly, left- and right-handers did not show the same pattern of results and the pattern for left-handers did not correspond to a simple reversal of the pattern for right-handers. While both groups showed evidence of visual enhancement when their dominant hand was near the display, their performance differed when their non-dominant hand was present (both alone and accompanied by their dominant hand). As we discuss below, these findings are consistent with the notion that visual processing in perihand space is determined by how people use each of their hands [11], [13].

For unimanual conditions, right-handers as well as left-handers showed enhanced visual sensitivity when their dominant hand was near the display, as would be expected [7], [10]. However, unlike right-handers, left-handers also showed a facilitation effect for their non-dominant hand. This finding is consistent with how left-handers control their actions. As already alluded to above, when people are free to choose a hand for precision grasping, right-handers clearly prefer their dominant hand, whereas left-handers use each hand equally often [12]. This suggests that the left-handers’ non-dominant hand has more action potential than for right-handers.

For the bimanual condition, right-handers showed slightly better visual sensitivity (but only numerically) when both their hands were near the display than when only their dominant hand was present. A similar pattern was obtained by Tseng and Bridgeman [7], who found better change detection in their both-hands condition than in their right-hand condition. They attributed their findings to a potential increase in attentional demands in bimanual situations, which could result in a form of nonlinear summation in the both-hands conditions. However, from an action-control perspective it is also important to consider how the potential for action changes in bimanual situations. For example, several studies have shown that each arm is specialized for certain aspects of motor control [27] resulting in a complementary role for the two arms [28]. Thus, the left hand, which is used less alone in right-handers [12], becomes more relevant when combined with the right hand and thereby increases the potential for forthcoming action. This increase in action potential is what could underlie the both-hands effect in right-handers.

However, for left-handers, visual sensitivity in the both-hands condition was similar to that in the non-dominant hand condition. The fact that the facilitation effect in the both-hands condition didn’t reach significance for left-handers probably reflects a slight power problem. More importantly, the difference between right- and left-handers with regards to the both-hands condition is consistent with the way people allocate attention in bimanual reaching. Buckingham, Main and Carey [29] observed that right-handers had more difficulty to inhibit a tactile cue to their right than their left hand before a bimanual reach, which indicates that they have an attentional bias toward their dominant hand. Left-handers exhibited no such bias. The lack of a strong left-right bias in left-handers suggests that they are potentially faced with more of a choice when it comes to allocating attention to each of their hands in bimanual situations. This could lead to a competition between the hands that would result in a form of interference effect for left-handers in the both-hands condition.

In conclusion, right- and left-handers manually interact with their environment in different ways and this seems to determine how visual sensitivity is enhanced near their hands. In relation to previous studies [7], [11], our findings further demonstrate that visual processing in perihand space is closely tied to potential forthcoming actions, even when the hands just happen to be there.
